# Insight into the Growth Mechanism of Low-Temperature Synthesis of High-Purity Lithium Slag-Based Zeolite A

**DOI:** 10.3390/ma17030568

**Published:** 2024-01-25

**Authors:** Li Li, Shicheng Xu, Ze Liu, Dongmin Wang

**Affiliations:** School of Chemical & Environmental Engineering, China University of Mining & Technology, Ding No. 11, Xueyuan Road, Haidian District, Beijing 100083, China; lily930622@163.com (L.L.); 18810596373@163.com (S.X.); wangdongmin@cumtb.edu.cn (D.W.)

**Keywords:** lithium slag, zeolite A, low-temperature, high-purity, growth mechanism

## Abstract

The utilization of lithium slag (LS), a solid waste generated during the production of lithium carbonate, poses challenges due to its high sulfur content. This study presents a novel approach to enhancing the value of LS by employing alkali fusion and hydrothermal synthesis techniques to produce zeolite A at low temperatures. The synthesis of high-purity and crystalline lithium-slag-based zeolite A (LSZ) at 60 °C is reported for the first time in this research. The phase, morphology, particle size, and structure of LSZ were characterized by XRD, SEM, TEM, N_2_ adsorption, and UV Raman spectroscopy, respectively. High-purity and crystalline zeolite A was successfully obtained under hydrothermal conditions of 60 °C, an NaOH concentration of 2.0 mol/L, and a hydrothermal time of 8 h. The samples synthesized at 60 °C exhibited better controllability and almost no byproduct of sodalite occurred compared to zeolite A synthesized at room temperature or conventional temperature (approximately 90 °C). Additionally, the growth mechanism of LSZ was elucidated, challenging the traditional understanding of utilization of lithium and enabling the synthesis of various zeolites at lower temperatures.

## 1. Introduction

Zeolites are aluminosilicate compounds with a three-dimensional structure, wherein oxygen atoms are shared between aluminum and silicon tetrahedral frameworks, resulting in a highly regular and open porous structure [1,2]. Among various zeolites, zeolite A is widely used in industry as an ion exchanger, adsorbent, and catalyst owing to its pore diameter (0.42 nm) and low silica characteristics [3,4]. The traditional synthesis of industrial zeolites uses NaAlO_2_ and Al (OH)_3_, SiO_2_ and Na_2_SiO_3_, and NaOH or KOH as aluminum sources, silicon sources, and alkali sources, respectively. But the raw material cost is relatively high. To reduce costs and solve environmental problems, researchers focused on industrial solid wastes as raw materials for the synthesis of zeolites, such as fly ash, paper sludge, kaolin, rice husk ash, coal gangue [5,6], and so on. The chemical composition of certain industrial solid waste is extremely similar to that of zeolite, providing the possibility for the synthesis of zeolite.

Using a sulfuric acid method to produce lithium carbonate generates a large amount of lithium slag. At present, the utilization of lithium slag in construction materials, such as cement and concrete, remains limited in scope [7,8]. Unfortunately, lithium slag contains more sulfur and usually limits the performance of cement-based materials. Removing sulfur from lithium slag requires significant costs and effort. Lithium slag is an ideal raw material for zeolite synthesis due to it being rich in silicon and aluminum phases and not being limited by sulfur. In lithium slag, the total content of SiO_2_ and Al_2_O_3_ is more than 80%. According to the analysis of its chemical composition, lithium slag is a great source of Si and Al, providing the basis for the synthesis of a pure zeolite A phase. Low content of CaO (only about 5%) is also a major advantage of synthesizing zeolite from lithium slag. It was first reported by Chen et al. [9] that zeolite NaX was synthesized from lithium slag. The crystallinity of zeolite NaX was as high as 94.31% and the adsorption capacity approached that of commercial zeolite. According to Lin et al. [10], lithium slag was used to prepare co-crystalline zeolite FAU/LTA-0 by the hydrothermal method that utilized mother liquid. It was directly used as a raw material by Huang et al. [11] to synthesize NaX zeolite using a hydrothermal and microwave-assisted method, and the zeolite had smaller grains (0.5–0.8 μm) and good stability. Wang et al. reported that zeolite A with excellent crystallinity can be synthesized under hydrothermal conditions using lithium slag activated by the new mild alkali fusion method [12]. The hydrothermal synthesis of zeolite A was generally carried out at a temperature of 90 to 120 °C for several hours. Previous research [13,14] indicated that excess energy was consumed under conventional hydrothermal synthesis temperatures (90~110 °C), and the hydroxy sodalite phases were formed along with the crystallization time being further prolonged. In the past decades, zeolite A was successfully obtained at room temperature [15,16,17,18]. Synthesis of zeolites at room temperature requires 48–72 h or even longer, and the grain size is difficult to control. Hence, it is necessary to develop a new route of zeolite A synthesis to improve purity and inhibit the formation of sodalite effectively. 

In this study, lithium-slag-based zeolite A of high purity and crystallinity was synthesized at 60 °C for the first time. Experimental parameters, such as temperature, NaOH concentration, and time, were examined to determine their impact on lithium-slag-based zeolite A and, in particular, reveal the possible growth mechanism of lithium-slag-based zeolite A. 

## 2. Experimental

### 2.1. Raw Materials

Lithium slag (LS) was collected from Lithium Industry Co. Ltd., Deyang, China. The chemical composition of LS includes SiO_2_ (58.27 wt %), Al_2_O_3_ (23.75 wt %), SO_3_ (9.06 wt %), CaO (5.81 wt %), Fe_2_O_3_ (1.36 wt %), and others (1.75 wt %). Sodium hydroxide (NaOH, AR) and sodium meta-aluminate (NaAlO_2_, AR) were provided by Beijing Chemical Co., Ltd., Beijing, China and Macklin Chemical Co., Ltd., Shanghai, China, respectively. Moreover, all solutions were produced using lab-made deionized water.

### 2.2. Preparation of Lithium-Slag-Based Zeolite A

Lithium-slag-based zeolite A (LSZ) specimens were prepared as follows: firstly, 7.5 g NaOH powder and 5 g LS were mixed and calcined at 650 °C for 2 h. Then, the calcined mixture was cooled at room temperature and NaAlO_2_ was added to adjust the SiO_2_/Al_2_O_3_ ratio. A total of 100 mL of deionized water was added to the mixture while stirring continuously. After stirring for 0.5 h and aging for 2 h, the resulting slurry was kept at 30~90 °C for 1~24 h in the hydrothermal reaction vessel. A final step involved repeatedly washing the synthetic zeolite samples in distilled water until the pH reached about 7–8 and drying at 80 °C.

### 2.3. Characterization

X-ray fluorescence spectroscopy (XRF, PANalytical B.V., Almelo, The Netherlands) was used to determine the chemical composition of lithium slag. Analysis of the crystalline phases of the LSZ was performed using X-ray diffraction (XRD, Smartlab 9000, Rigaku, Tokyo, Japan) with Cu Kα radiation at a scanning speed of 5°/min between 2θ = 5° to 50°. The surface morphology, particle size, and elemental content distribution were examined through the utilization of scanning electron microscopy (SEM, JSM-7001F, JEOL, Tokyo, Japan) and high-resolution transmission electron microscopy (HRTEM, JEM-2100F, JEOL, Tokyo, Japan). UV Raman spectroscopy (Horiba Scientific LabRAM HR Evolution, Villeneuve d’Ascq, France) was utilized in the range of 100–1250 cm^−1^ and was highly sensitive to the vibration of structural units, rendering it convenient for structural analysis. Furthermore, the specific area and size distribution of the pores were measured by N_2_ adsorption–desorption (ASAP 2020HD88, Micromeritics, GA, USA).

## 3. Results and Discussion

### 3.1. Characterization of Raw Materials and LSZ

**Figure 1:**
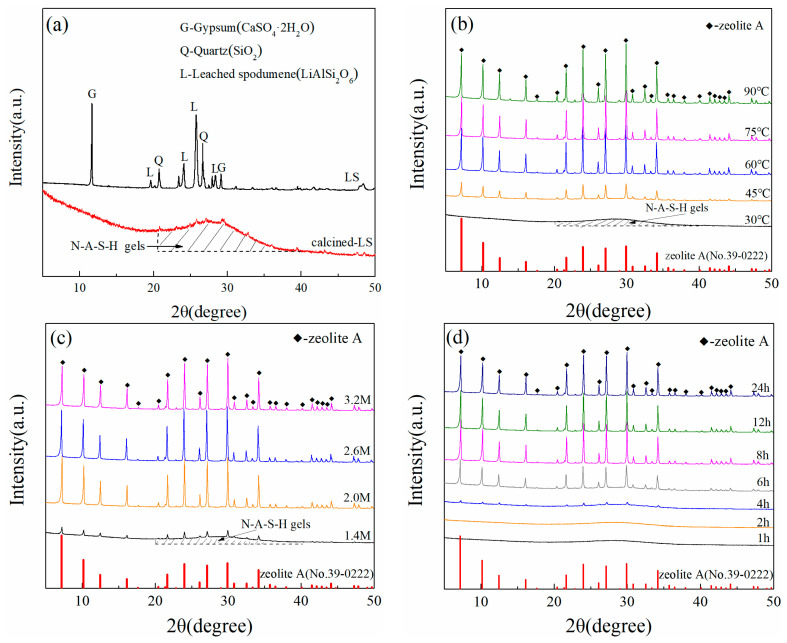
XRD patterns of raw materials and different preparation conditions of zeolite: (**a**) raw materials; (**b**) different temperatures; (**c**) different alkalinity; (**d**) different time.

Figure 1 shows the XRD patterns of raw materials and lithium-slag-based zeolite prepared in different conditions. As shown in Figure 1a, there is a great difference in lithium slag before and after calcination. The glass phase of lithium slag can be dissolved and the inert crystal phase structure destroyed with alkali during the calcination process, further enhancing the activity of lithium slag. The XRD pattern of lithium slag showed gypsum, quartz, and leached spodumene as the main phases. The crystalline substance of lithium slag disappeared completely and was replaced by amorphous N-A-S-H gels due to the alkali fusion step (650 °C for 2 h), which can easily dissolve those components and is beneficial to the nucleus formation process and crystal growth process of zeolite later. The existence of quartz and leached spodumene crystals will have a serious impact on the synthetic purity of lithium-slag-based zeolite. Hence, it is necessary to remove quartz crystals or transform quartz and leached spodumene into amorphous N-A-S-H gels. The quartz and leached spodumene successfully became N-A-S-H gels, which have higher activity and were an ideal raw material for LSZ hydrothermal synthesis at low temperatures. The single-factor control variable method was adopted and experimental results are presented in Table 1.

The XRD patterns indicating the crystallization of samples prepared at different temperatures (30, 45, 60, 75, and 90 °C) after 12 h at an alkalinity of 2.6 mol/L are shown in Figure 1b. Sample T-30 remained as amorphous N-A-S-H gel and did not form zeolite crystal at 30 °C. As the crystallization temperatures increased from 45 °C to 90 °C, characteristic peaks of zeolite A crystals were gradually observed, implying the reaction temperature strongly affects the nucleation process and crystal growth process. Lin et al. [19] pointed out that the reaction temperature strongly affects the nucleation process and crystal growth process. It is clearly seen that the diffraction intensities of the peaks become stronger with the increase in hydrothermal temperature from 30 to 90 °C.

However, the presence of a zeolite A crystalline structure in sample T-45, as compared to the zeolite A standard PDF card (No. 39-0222), was observed, albeit with low intensity, indicating an incomplete crystallization process. Sample T-60 shows better crystallinity, increasing slightly with the increase in temperature, suggesting that zeolite A crystallization was almost complete. Hence, 60 °C is selected as the appropriate crystallization temperature to be used in other synthesis procedures. 

To investigate the influence of alkalinity on the phase and structure of the products, zeolite A was synthesized at an alkalinity of 1.4, 2.0, 2.6, and 3.2 mol/L by adjusting the concentration of sodium hydroxide (NaOH) solution. Generally, the alkalinity of zeolite prepared from chemical reagents is about 2.6 mol/L. Because there are many impurities in solid waste, the alkalinity required will be increased accordingly. The conventional hydrothermal synthetic alkalinity of solid-waste-based zeolite is approximately 3.0 mol/L. For example, the synthetic alkalinity of lithium-slag-based zeolite X is 3.75 mol/L by Chen et al. [9]. Liu et al. found that a large number of fly-ash-based zeolite A is formed at 2.6 mol/L of NaOHaq [20]. The XRD patterns indicating the effect of different levels of alkalinity on the preparation of zeolite at 60 °C for 12 h are provided in Figure 1c. Zeolite A starts to form when the NaOHaq concentration is 1.4 mol/L, but the diffraction patterns of zeolite A is weak due to the Si and Al not fully dissolving in the lithium slag. The typical and complete peak of zeolite A appeared when alkalinity was 2.0 mol/L without an additional phase, and the intensity of XRD peaks did not further enhance when the NaOHaq concentration rose from 2.0 mol/L to 3.2 mol/L. It is important to note that alkalinity plays an essential role in the synthesis of zeolite A [21]. Alkalinity would affect the dissolution rate of silicon and aluminum in lithium slag, and adjust the polymerization state and distribution of the polymer in the gel system. The initial substances need to be depolymerized and rearranged through OH^−^ ions. Crystal substances such as mullite and quartz in the lithium slag convert to soluble aluminosilicate through an alkali fusion process, corresponding to alkalinity, which is also reduced.

Furthermore, the crystallization time of zeolites under conditions of 60 °C and 2.0 mol/L was investigated. The XRD patterns of samples crystallized from 1 h to 24 h can be seen in Figure 1d. It shows no crystal structure after 12 h of hydrothermal treatment. Some weak peaks of zeolite A were distinguishable after 4 h, but the sample t-4 still included a large quantity of amorphous material. The peak intensity of zeolite A enhanced significantly with the hydrothermal increase at 6 h, and well-crystallized zeolite A was obtained after 8 h. However, extending the hydrothermal duration to 12 h and 24 h does not appear to have any impact on the intensity of zeolite A peaks, suggesting that the crystallization process of the sample was nearly finished within 8 h.

**Figure 2:**
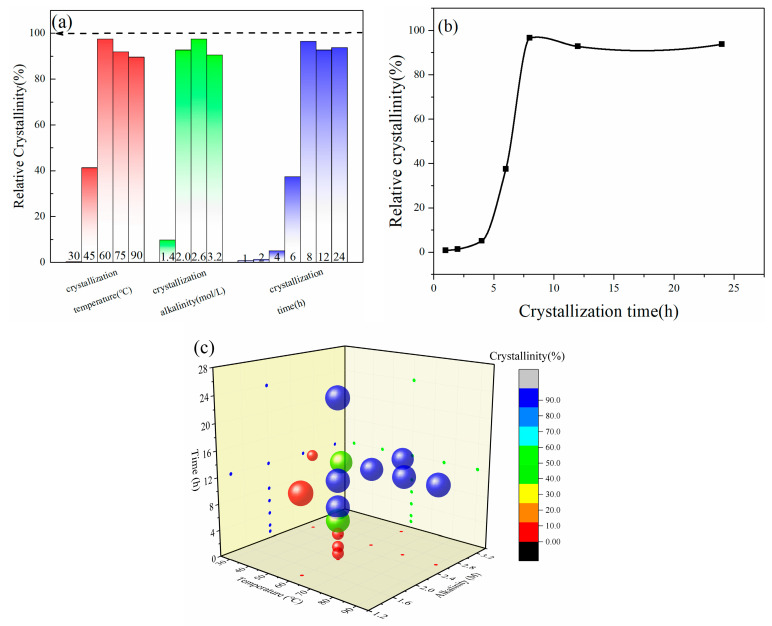
Relative crystallinity chart: (**a**) crystallization column graph; (**b**) crystallization curve; (**c**) three-dimensional scatterplot of three factors.

The relative crystallinity of samples as calculated by Jade 6 software is shown in Figure 2a, referring to the crystallinity of commercial zeolite A as 100%. The relative crystallinity of samples under hydrothermal synthesis conditions between 30 °C and 90 °C are 0.3%, 41.3%, 97.5%, 91.9%, and 89.6%. Generally, the crystallization process of zeolite includes three stages: precursor formation (induction period), nucleation, and crystal growth [22,23]. From the crystallization curve in Figure 2b, it can be seen that the relative crystallinity of the samples is nearly zero at 0~2 h, which is the induction period for the growth of LSZ. The curve rises rapidly during 2~8 h with a large slope, suggesting that this period is the main growth period of LSZ. There was no significant change in relative crystallinity after 8 h, indicating that the crystal growth of LSZ has been finished. The three-dimensional scatter plot (Figure 2c) clearly shows the effect of synthesis temperature, alkalinity, and time on LSZ. As such, we can find that 60 °C is an ideal synthesis temperature for LSZ.

**Figure 3:**
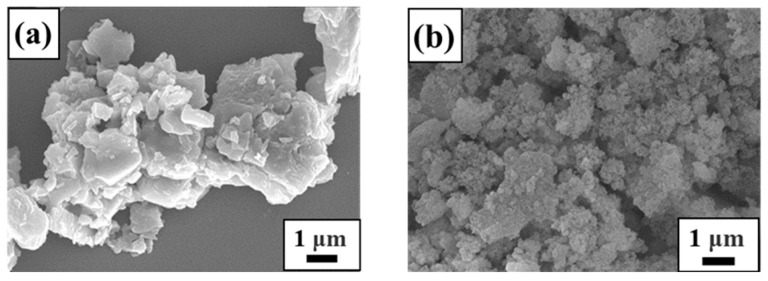
SEM morphology of raw materials: (**a**) lithium slag; (**b**) calcined lithium slag.

**Figure 4:**
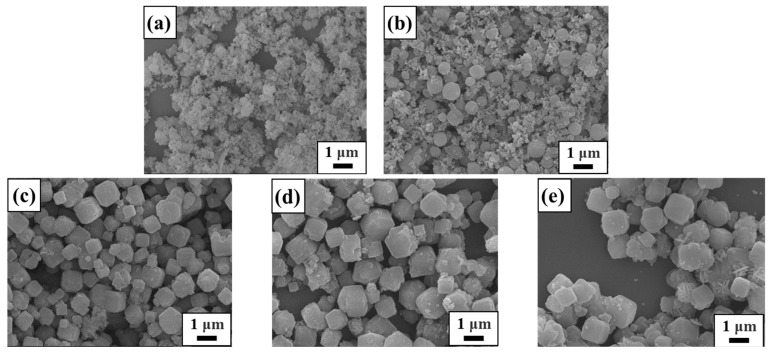
SEM images of samples obtained at different synthesis temperatures: (**a**) 30 °C; (**b**) 45 °C; (**c**) 60 °C; (**d**) 75 °C; (**e**) 90 °C.

**Figure 5:**
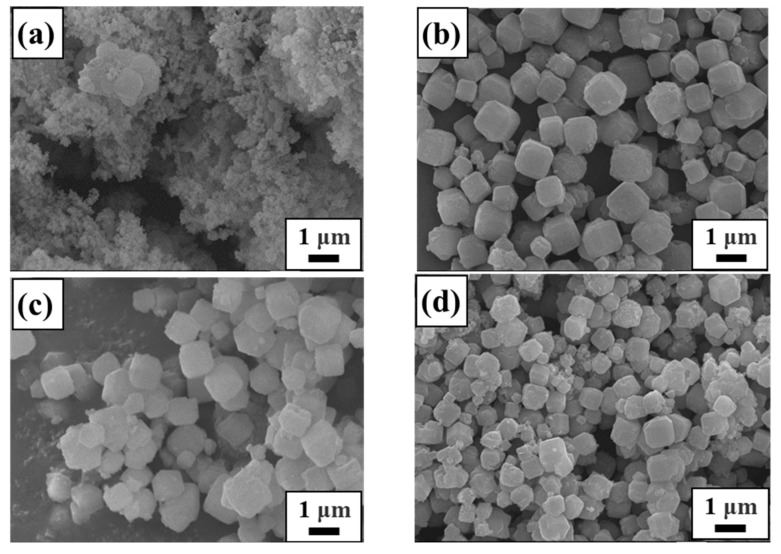
SEM images of samples obtained with different synthesis alkalinity values: (**a**) 1.4 mol/L; (**b**) 2.0 mol/L; (**c**) 2.6 mol/L; (**d**) 3.2 mol/L.

**Figure 6:**
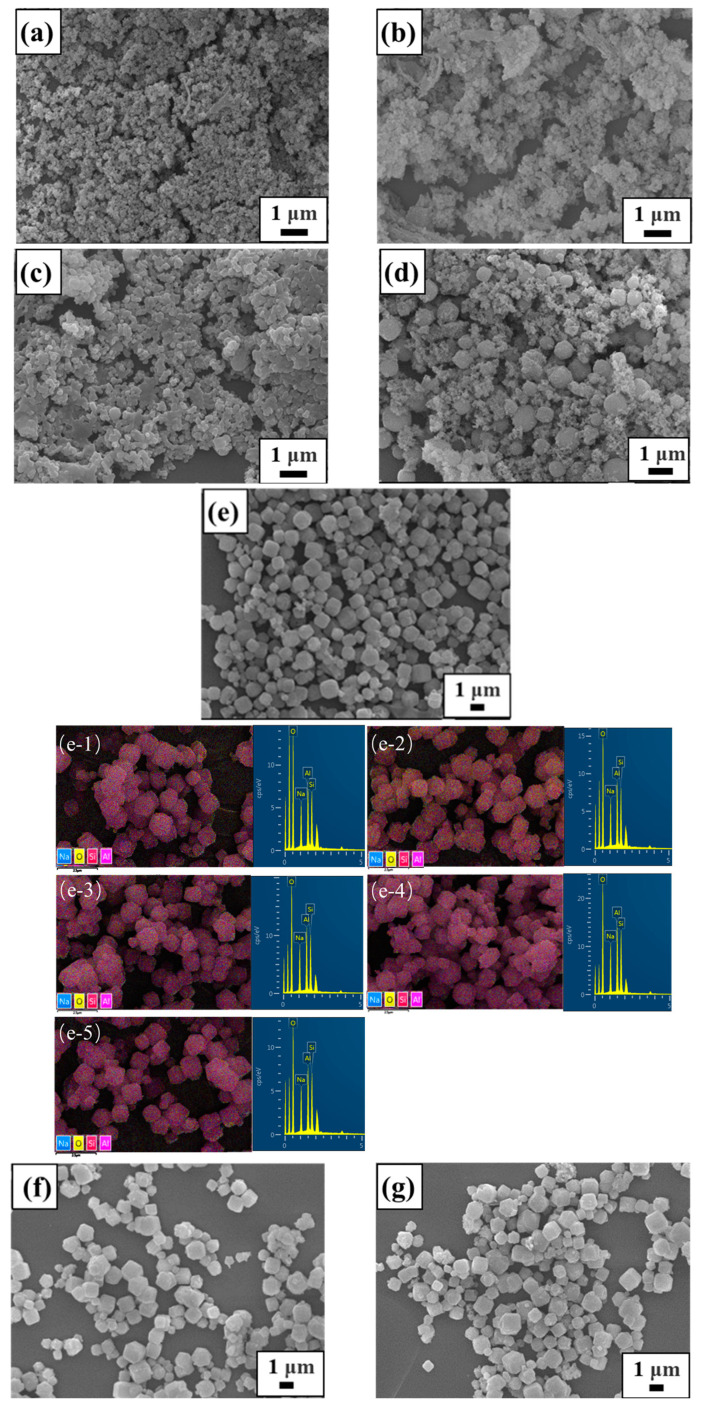
SEM images of samples obtained at different synthesis times: (**a**) 1 h; (**b**) 2 h; (**c**) 4 h; (**d**) 6 h; (**e**,**e-1**–**e-5**) 8 h; (**f**) 12 h; (**g**) 24 h.

The SEM images of raw materials and synthetic samples under different conditions are displayed in Figure 3, Figure 4, Figure 5 and Figure 6. From Figure 3, it is clearly seen that lithium slag becomes a loose and porous gel within alkali fusion, contrary to the massive and irregular polygonal-shaped crystals. Most of the minerals in the lithium slag have irregular shapes, which are formed by cooling under unstable thermal conditions. Sample T-30 remained gel state through 30 °C hydrothermal treatment and has no distinction from calcined lithium slag in Figure 4a. According to Figure 4b, the morphology of T-45 is presented as an approximately spherical crystal mixed with many gel particles, suggesting zeolite A has already been generated. This result is in agreement with the XRD patterns above. There are still some precursors in the product that did not participate in the reaction when the temperature was low. At this moment, the product has an approximately cubic shape with unclear edges and different sizes, and many irregular amorphous substances. When the hydrothermal temperature rises to 60~75 °C, amorphous gels disappear and the product shows a regular cubic morphology while the relative crystallinity reaches about 95%. As the reaction temperature continued to rise to 90 °C, a small amount of zeolite A in the product was transformed into sodalite. Compared with XRD results, it is possible that the peak of zeolite A covered up the characteristic peak of sodalite due to the strong peak of zeolite A. Considered comprehensively, it is suitable to synthesize zeolite A under the mild conditions of a hydrothermal temperature of 60 °C. 

Using a 60 °C hydrothermal temperature and 12 h hydrothermal time, the effect of alkalinity on synthetic zeolite was investigated. The morphologies of samples obtained with different synthesis alkalinity values and time are shown in Figure 5 and Figure 6, respectively. It can be seen that in sample A-1.4 only a few gel particles grow to zeolite A and its surface is covered by aluminosilicate gels. A great deal of zeolite A crystals appear in a cube crystalline shape with a size of ~1 μm using 2.0 mol/L alkalinity. The structure of zeolite A is connected by four-membered rings with oxygen bridges, and it is kept stable in a system with slightly lower alkalinity. The product still maintained the shape of a cube and the size of zeolite A was also about 1 μm when the concentration of NaOHaq increased to 2.6 mol/L. However, the existence of sodalite can be found, as observable in Figure 5d, which will reduce the regularity of the cubic morphology of the product, and some fine irregular substances appear at the same time. There is a possibility that the morphology is destroyed because the alkalinity is too high, causing zeolite A to transform into other crystals. Therefore, 2.0 mol/L is the ideal alkalinity and is lower than the traditional synthetic alkalinity of solid-waste-based zeolite.

The effect of hydrothermal time on the product was analyzed under the conditions of hydrothermal temperature of 60 °C and hydrothermal alkalinity of 2.0 mol/L. As can be seen in Figure 1d and Figure 6, the crystalline phase of zeolite A was not formed within 0~2 h of hydrothermal time, and amorphous gels in the sample did not change. With the increase in hydrothermal time to 4 h, small-particle gels grew, and a small amount of large spherical particles can be observed. As the hydrothermal time continues to increase to 6 h, the amorphous material is greatly reduced and the cubic morphology of the product is gradually regular. At the same time, it can be inferred that 4~6 h is the rapid growth period of zeolite A. After the hydrothermal time reached 8 h, the cubic morphology of zeolite A was clearer, with clear edges and a smooth surface, indicating that the crystal growth was completed. After 8 h, the morphology and particle size of the product did not change significantly, which demonstrated that the crystal did not grow further. Therefore, 8 h is an ideal time for the synthesis of zeolite A under low-temperature and low-alkali conditions. The product synthesized at 8 h was also analyzed by EDS (as displayed Figure 6e1–e5), which showed that the atomic ratio of Na:Al:Si = 1.07:1:0.95 (average of five different areas) was close to 1:1:1, matching the chemical composition of zeolite A(Na_12_(Al_12_Si_12_O_48_)·27H_2_O). If the crystallization time continues to extend, metastable zeolite A may be transformed into dense sodalite and other crystalline phases, affecting the purity of the product.

**Figure 7:**
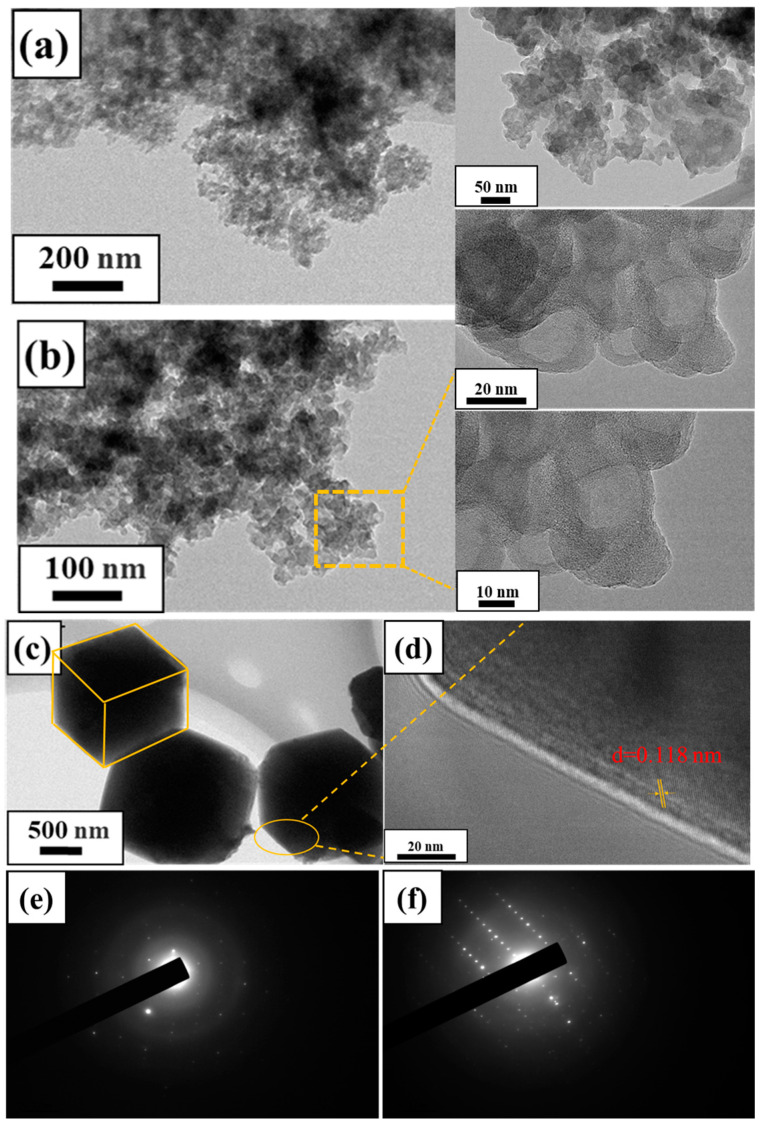
TEM images of raw materials and samples: (**a**,**b**) calcined lithium slag; (**c**–**f**) zeolite, 60 °C, 2.0 mol/L, 8 h.

Figure 7a–f displays the images of the samples recorded using HRTEM, while the microstructure of the samples was examined through the utilization of HRTEM. The calcined lithium slag and zeolite are shown in Figure 7. The morphology and size of zeolite crystals can be directly determined using both SEM and TEM, and it can be seen from Figure 7a,b that the lithium slag crystals disappear and no lattice striations exist after calcination and washing. It shows an amorphous gel state, which is also the main phase of the lithium slag active source and provides great convenience for the subsequent low-temperature and low-alkali synthesis. Figure 7c illustrates the cubic morphology of the samples, as well as near uniform size distributions with good crystallinity. Figure 7d shows the lattice fringing of LSZ with a calculated d-spacing value of 0.118 nm, corresponding to the (221) plane of the A-type zeolite structure. Figure 7e,f shows selected area electron diffraction (SAED) plots showing diffraction patterns corresponding to the (h k l) planes of (111), (210), (321), and (322), which are in good agreement with the XRD results of LSZ. This notwithstanding, it is not difficult to find that the zeolite crystals synthesized at 60 °C are regular in shape and uniform in size, with particle size around 1 μm and no impurity particles attached to the surface. In addition, a few square sodium stones were produced, indicating that this synthesis condition is better than the conventional synthesis temperature of 90 °C. 

**Figure 8:**
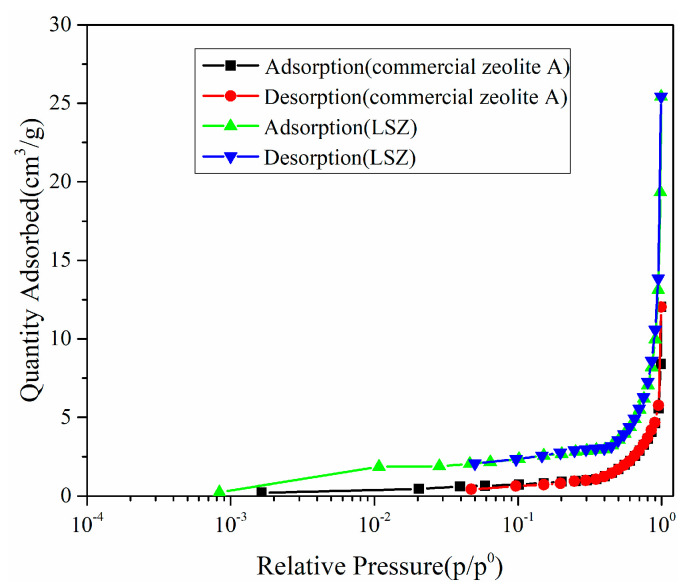
The semi-logarithmic adsorption isotherms of commercial zeolite A and LSZ.

The surface structure parameters of commercial zeolite A and LSZ are shown in Figure 8 and Table 2. The samples need to be degassed at 120 °C for 12 h before the N_2_ adsorption test. As shown in Figure 8, commercial zeolite A and LSZ represent a type I isotherm (according to the BDDT (Brunauer–Deming–Deming–Teller) methodology). Type I isothermal curves are typical isothermal curves for adsorption and desorption of microporous materials. From Table 2, it can be observed that the specific surface areas of commercial zeolite A and LSZ were 2.6 and 7.0 m^2^/g, respectively, according to the multi-point BET model, and the specific surface area of LSZ was more than twice that of commercial zeolite A. The zeolite particles synthesized at a low temperature are small in size and prone to agglomeration, which may lead to a decrease in specific surface area. The significant increase in surface area and pore volume of synthesized LSZ indicates that the adsorption capacity of LSZ synthesized by the alkali fusion low-temperature hydrothermal method is significantly improved. 

### 3.2. The Growth Process of LSZ

**Figure 9:**
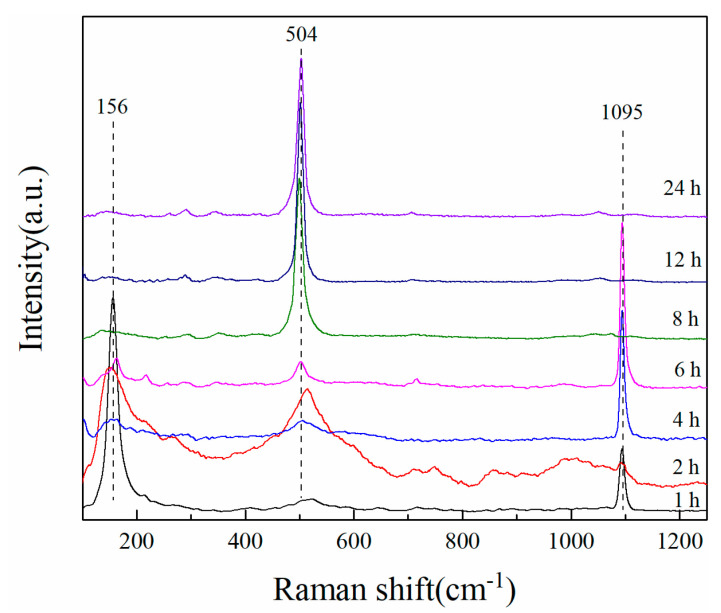
Ultraviolet Raman spectra of samples with different hydrothermal times.

While XRD patterns and SEM images are capable of illustrating the progressive alterations in the zeolite growth process, they are insufficient in elucidating the modifications occurring in the fundamental structural units during zeolite formation. Therefore, it is necessary to study such with the help of UV Raman spectroscopy, which is highly sensitive to the vibration of structural units. Figure 9 shows the UV Raman spectra of products at different hydrothermal times. In the hydrothermal reaction process of 1~4 h, there are overlapping peaks in the vicinity of 500 cm^−1^, confirming that the system contains a large number of four-membered ring structure units [24] and the amorphous gels are the main phase still in the product. The most significant change in the UV Raman spectrum occurred after 8 h of crystallization. With the extension of the hydrothermal time, the peak at 504 cm^−1^ became sharp and strong, explained by the degree of order gradually increasing, where the strongest peak was attributed to the bending vibration of the quaternary silicon–aluminum ring in the zeolite. The spectral peak at 1095 cm^−1^ belongs to the antisymmetric stretching vibration of the T-O (T = Si, Al) bond [25]. After the complete formation of zeolite A, the spectral peak at 1095 cm^−1^ disappeared. 

In summary, the LSZ growth process is divided into three steps. The first stage is the primary gel dissolution, where the polymeric silica–alumina species undergo depolymerization. The second stage is the interaction of the depolymerized mono-polymeric silica–alumina species, which are connected to form the secondary structural units required for the zeolite skeleton. At the end of this process, zeolites are formed, with secondary units interacting, rearranging, assembling, and then joining to form the zeolite skeleton.

**Figure 10:**
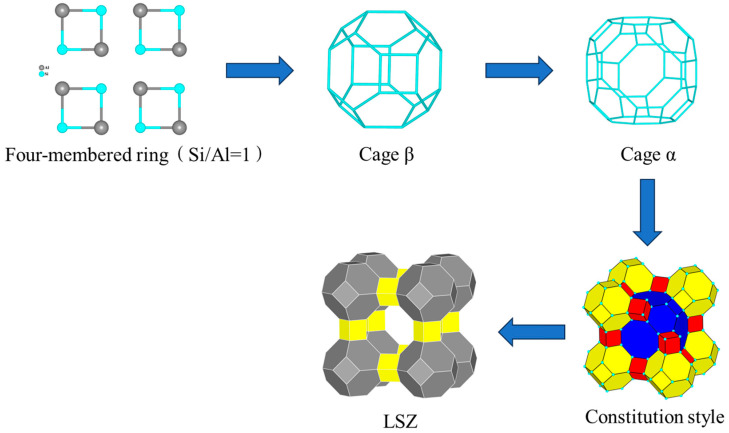
Schematic diagram of the growth process of LSZ.

To gain further insight into the growth mechanism of zeolite, the growth process of LSZ is analyzed in Figure 10. The gel initially consists of a significant quantity of Si/Al = 1 quaternary rings during the early stages of crystallization. Over time, these quaternary rings become interconnected, resulting in the formation of β-cages. As the crystallization process advances, both β-cages and quaternary rings persistently connect to generate α-cages within the nucleus center. Subsequently, β-cages within the nucleus center continue to interconnect, leading to the creation of novel α-cages that undergo continuous connection until ultimately forming A-type zeolite crystals [26].

## 4. Conclusions

In this study, the conversion of lithium slag into amorphous gels was achieved through pretreatment using alkali fusion at a temperature of 650 °C for 2 h. The impact of hydrothermal temperature, alkalinity, and time on the synthesis of alkali fusion lithium slag and its resulting crystalline end products was investigated. The optimal conditions for the synthesis of LSZ were found to be a low hydrothermal temperature of 60 °C, an NaOH concentration of 2.0 mol/L, and a hydrothermal duration of 8 h. A high crystallinity of 96.5% was achieved for LSZ. Analysis using XRD and SEM confirmed the successful synthesis of LSZ with a well-defined crystalline phase. In the course of zeolite growth, the XRD patterns, SEM and TEM images, UV Raman spectroscopy, and relative crystallinity exhibited progressive alterations. It was observed that the generation of by-product sodalite significantly diminished under conditions characterized by low temperatures and low alkali concentrations, as opposed to those at 90 °C and 2.6 mol/L.

## Figures and Tables

**Table 1:** Experiment designs with levels, crystallinity, and reaction conditions of samples.

Samples Name	Crystallization Temperature (°C)	Crystallization Alkalinity cNaOHaq (mol/L)	Crystallization Time (h)	Relative Crystallization Percent (%)	Remarks
Effect of crystallization temperature					
T-30	30	2.6	12	0.3	Amorphous gels
T-45	45	2.6	12	41.3	Zeolite A + amorphous gels
T-60	60	2.6	12	97.5	Zeolite A
T-75	75	2.6	12	91.9	Zeolite A
T-90	90	2.6	12	89.6	Zeolite A
Effect of alkalinity					
A-1.4	60	1.4	12	9.8	Zeolite A + amorphous gels
A-2.0	60	2.0	12	92.7	Zeolite A
A-2.6	60	2.6	12	97.5	Zeolite A
A-3.2	60	3.2	12	90.5	Zeolite A
Effect of crystallization time					
t-1	60	2.0	1	0.8	Amorphous gels
t-2	60	2.0	2	1.3	Amorphous gels
t-4	60	2.0	4	5.0	Zeolite A + amorphous gels
t-6	60	2.0	6	37.4	Zeolite A + amorphous gels
t-8	60	2.0	8	96.5	Zeolite A
t-12	60	2.0	12	92.7	Zeolite A
t-24	60	2.0	24	93.8	Zeolite A

**Table 2:** Surface structure parameters of commercial zeolite A and LSZ.

Sample	S_BET_/m^2^·g^−1^	Pore Volume/cm^3^·g^−1^
Commercial zeolite A	2.6	0.018
LSZ	7.0	0.039

## Data Availability

Data are contained within the article.
